# Incurred Sample Reanalysis: Time to Change the Sample Size Calculation?

**DOI:** 10.1208/s12248-019-0293-2

**Published:** 2019-02-11

**Authors:** Piotr J. Rudzki, Przemysław Biecek, Michał Kaza

**Affiliations:** 1 Pharmacokinetics Department, Pharmaceutical Research Institute, 8 Rydygiera Street, 01-793 Warsaw, Poland; 2 Faculty of Mathematics and Information Science, Warsaw University of Technology, 75 Koszykowa Street, 00-662 Warsaw, Poland

**Keywords:** incurred sample reanalysis (ISR), bioanalysis, hypergeometric distribution, bioanalytical method validation, bridging data

## Abstract

Reliable results of pharmacokinetic and toxicokinetic studies are vital for correct decision making during drug discovery and development. Thus, ensuring high quality of bioanalytical methods is of critical importance. Incurred sample reanalysis (ISR)—one of the tools used to validate a method—is included in the bioanalytical regulatory recommendations. The methodology of this test is well established, but the estimation of the sample size is still commented on and contested. We have applied the hypergeometric distribution to evaluate ISR test passing rates in different clinical study sizes. We have tested both fixed rates of the clinical samples—as currently recommended by FDA and EMA—and a fixed number of ISRs. Our study revealed that the passing rate using the current sample size calculation is related to the clinical study size*.* However, the passing rate is much less dependent on the clinical study size when a fixed number of ISRs is used. Thus, we suggest using a fixed number of ISRs, e.g., 30 samples, for all studies. We found the hypergeometric distribution to be an adequate model for the assessment of similarities in original and repeated data. This model may be further used to optimize the sample size needed for the ISR test as well as to bridge data from different methods. This paper provides a basis to re-consider current ISR recommendations and implement a more statistically rationalized and risk-controlled approach.

## INTRODUCTION

Reliable results of pharmacokinetic and toxicokinetic studies are vital for correct decision making during drug discovery and development. Thus, assuring high quality of bioanalytical methods is of critical importance. The American Association of Pharmaceutical Scientists (AAPS) and the US Food and Drug Administration (FDA) were the driving forces behind discussions on the bioanalytical method validation in the 1990s (1). Both AAPS and FDA are constantly involved in the evolution of bioanalytical requirements, including incurred samples reanalysis (ISR) (2,3). Professional organizations like the European Bioanalysis Forum have also presented their opinions on the test (4). Finally, the ISR was included in the bioanalytical regulatory recommendations by the European Medicines Agency (EMA) (5), the Health Canada (6), and the FDA (7). Although the ISR is now part of the regulatory documents, the topic is still under much discussion in the bioanalytical and pharmaceutical community (8–16).

One of the most debated aspects of the ISR test is the estimation of its sample size. Originally, Rocci *et al.* proposed a fixed number of ca. 20 samples, which was argued to detect a 20% difference for small molecules with 0.8 power and 0.05 type I error (17). The European Bioanalysis Forum agreed with Rocci *et al.* and suggested a fixed number of 20–50 samples per study (4), but did not justify it statistically. Enhanced statistical considerations and the simulations presented by Hoffman revealed that for accurate (bias = 0%) and precise (CV = 10%) methods, 40 samples allowed correct decision making (18). Thway *et al.* reviewed their studies on macromolecules and used simulations to investigate the influence of the method precision on the probability of passing the ISR test (19). They revealed that 40 samples are enough to pass a 30% difference criterion with a probability close to 1, whereas the precision did not exceed 15%. The Global Bioanalysis Consortium suggested, in turn, to reanalyze the fixed ratio of 5% of clinical samples that is equal to the ratio of quality control (QC) samples in a bioanalytical batch (9). More recently, Subraminiam *et al.* presented a comprehensive ISR simulation for small molecules using various combinations of precision and bias (10). It revealed that if 20 ISRs are analyzed and the method’s precision up to 15% is combined with the bias not exceeding 10%, then over 0.85 of the studies pass the ISR test. All these simulations contributed to the knowledge on how different factors influence the ISR sample size. They also suggest that for reproducible methods, only 40–50 samples are enough to meet the ISR passing rate over 99%. Yet, some of the assumptions seem questionable, like no bias (18,19) or a narrow concentration range (10,19). Moreover, we have recently suggested that the number of ISRs recommended by FDA and EMA (5,7) does not seem to be well matched with the acceptance criteria (12).

To illustrate the assumptions-free approach, let us gamble for a moment. Imagine an urn with green and red balls. Our goal is to guess if the ratio of green balls to the red ones is not lower than 2:1. How many balls do we need to pick to be confident that the ratio would be 2:1? Does our confidence depend on the number of balls in the urn or rather on the true ratio of the balls? Now, let us replace ball picking with a bioanalytical method, and green balls with the passed ISR pair. Then, we can rephrase the question: how many ISRs should we analyze to confirm the method’s reliability? Does it depend on the clinical study size or rather on the method’s performance itself? Based on the urn example, the hypergeometric distribution may help us answer these questions. This theoretical distribution is used in physics, biology, medicine, and chemistry, for example to study signal-to-noise ratios (20), as well as in the mass spectrometric identification of proteins (21,22), phospholipids (23), and elemental composition of unknown compounds (24). This is the first study that uses the hypergeometric distribution to evaluate the ISR sample size.

In this paper, we have aimed to provide researchers and regulators with a model for estimating and optimizing the number of ISRs needed to prove the reliability of a bioanalytical method. We have applied the hypergeometric distribution to evaluate ISR test passing rates in different clinical study sizes.

## MATERIALS AND METHODS

**Fig. 1:**
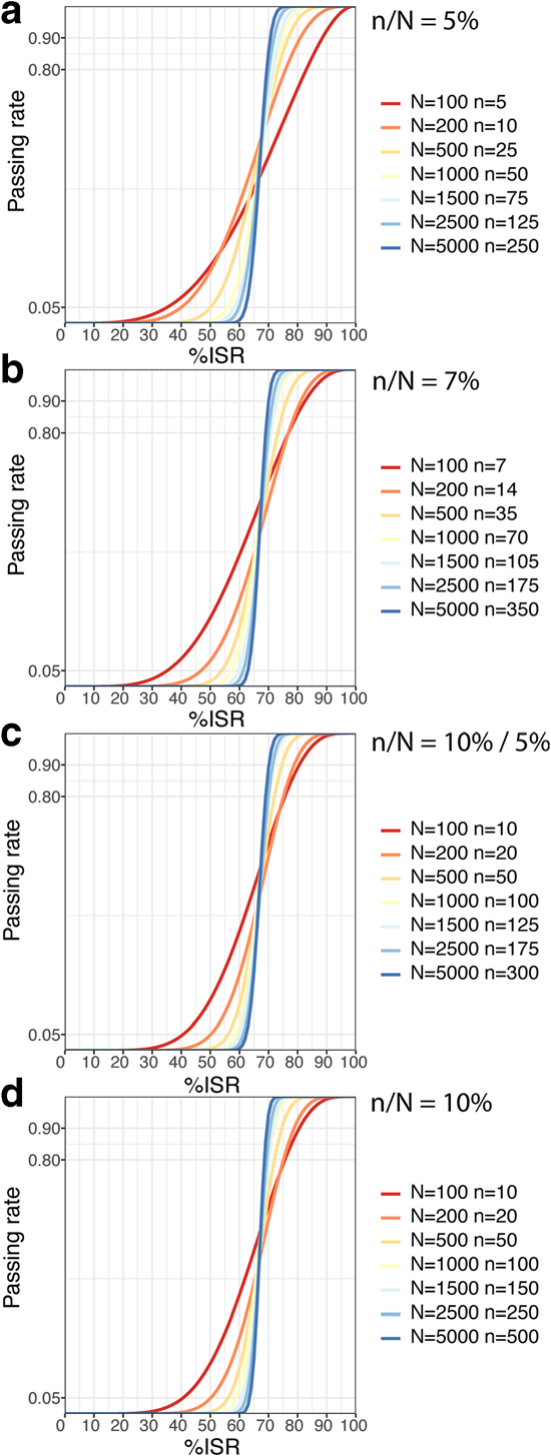
Cumulative distribution function for different study sample sizes (*N*), when the ratio of the number of ISRs to study the sample size (*n/N*) is fixed at 5% (**a**), 7% (**b**), 10% up to 1000 samples, and then 5% (**c**) and 10% (**d**)

**Fig. 2:**
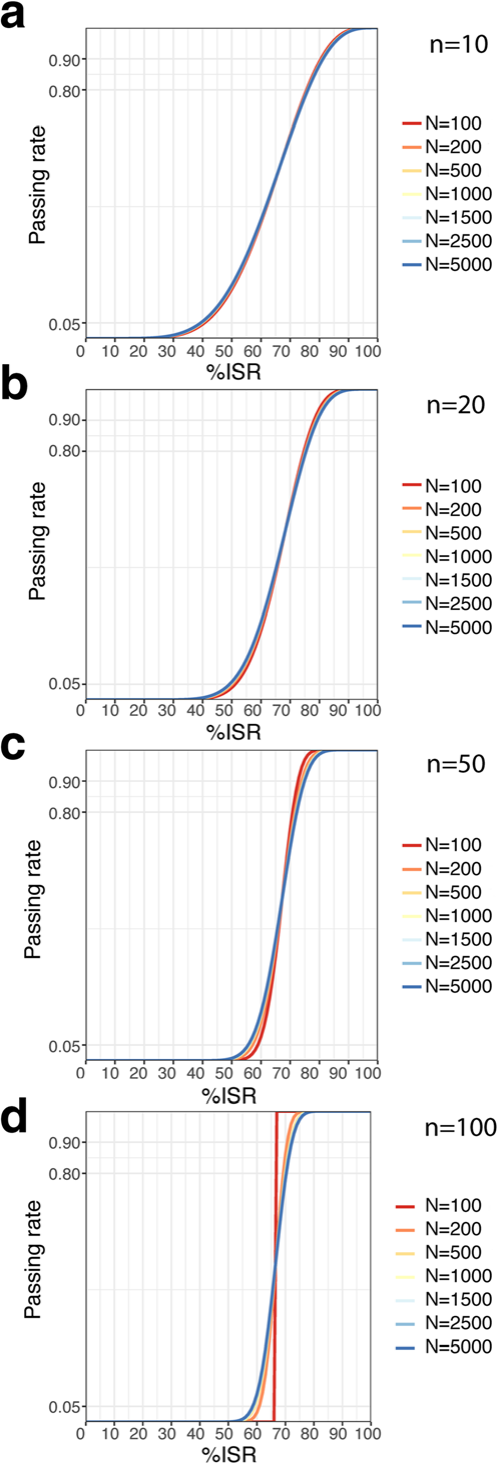
Cumulative distribution function for different study sample sizes (*N*), when the number of ISRs (*n*) is fixed at 10 (**a**), 20 (**b**), 50 (**c**), and 100 (**d**)

**Fig. 3:**
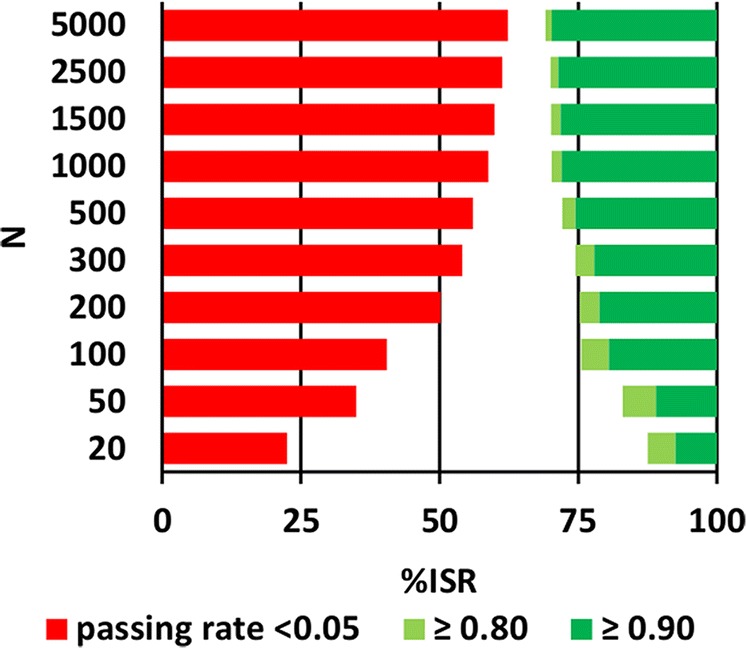
Differentiation of non-reproducible (red) and reproducible (green) methods for different study sample sizes (*N*) using the current regulatory ISR sample size (5,7)

**Fig. 4:**
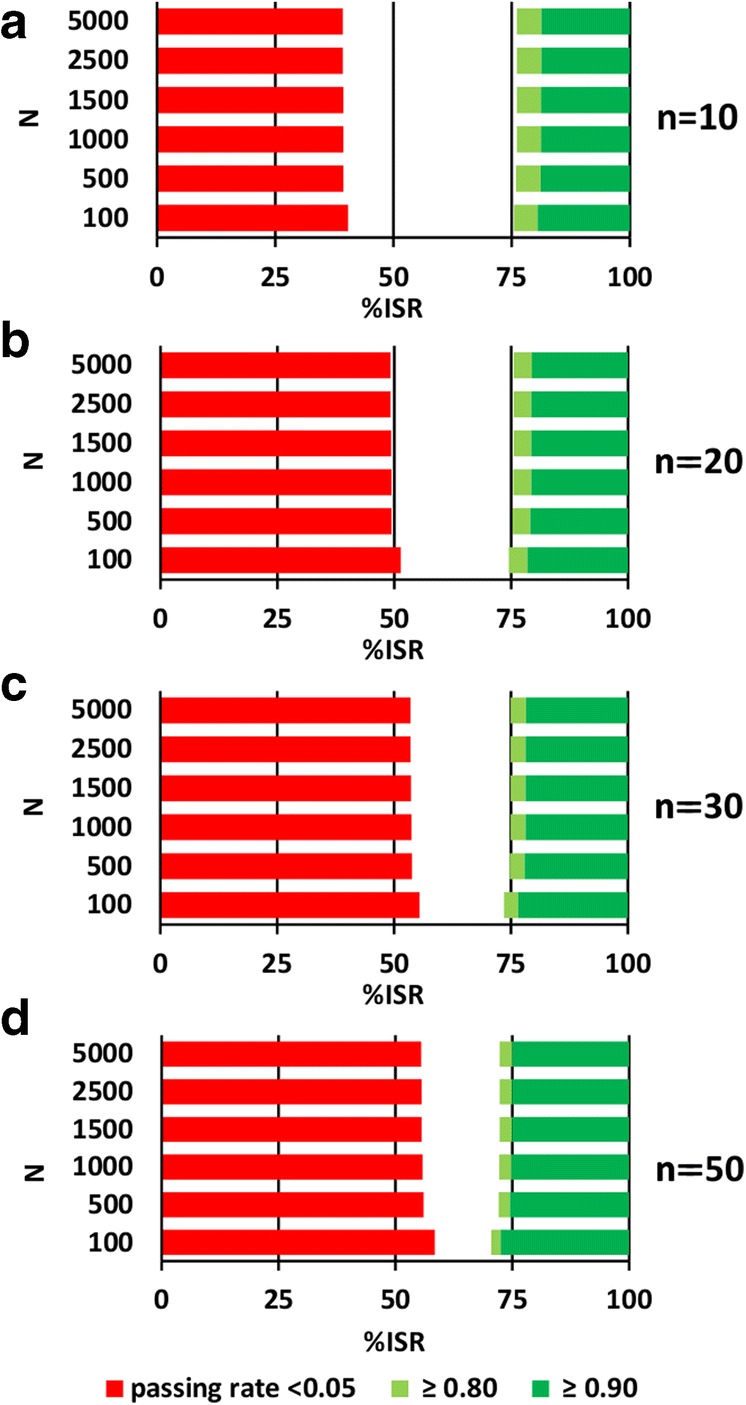
Differentiation of non-reproducible (red) and reproducible (green) methods for different study sample sizes (*N*), when the number of ISRs (*n*) is fixed at 10 (**a**), 20 (**b**), 30 (**c**), and 50 (**d**)

**Fig. 5:**
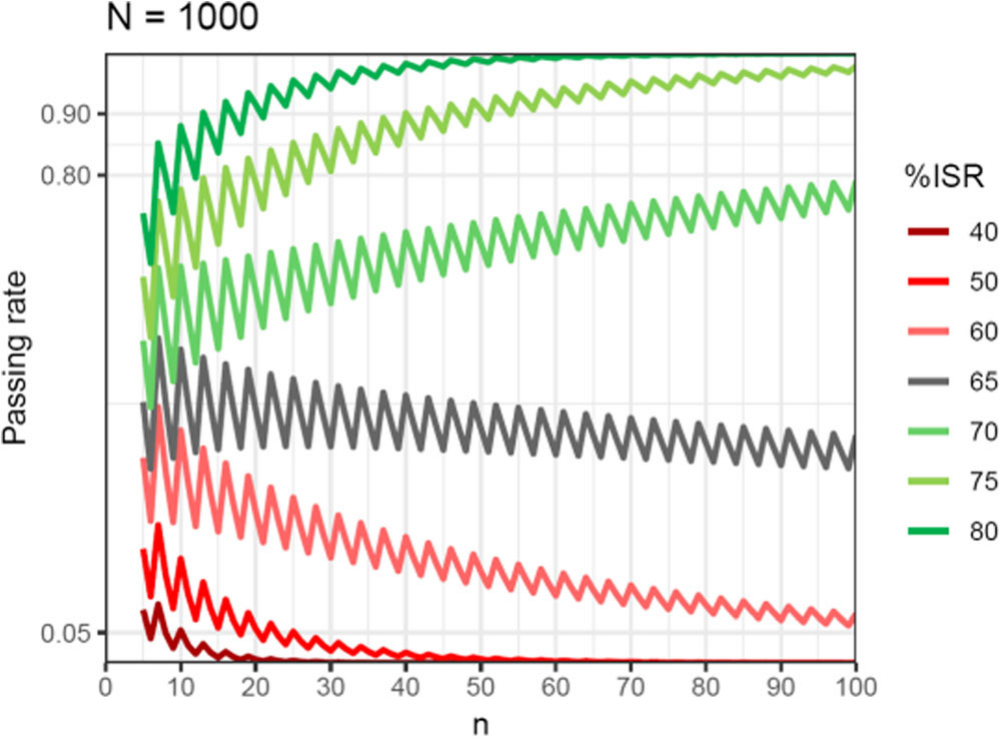
Passing rates for different %ISRs as a function of the number of ISRs (*n*) for the study sample size *N* = 1000

**Fig. 6:**
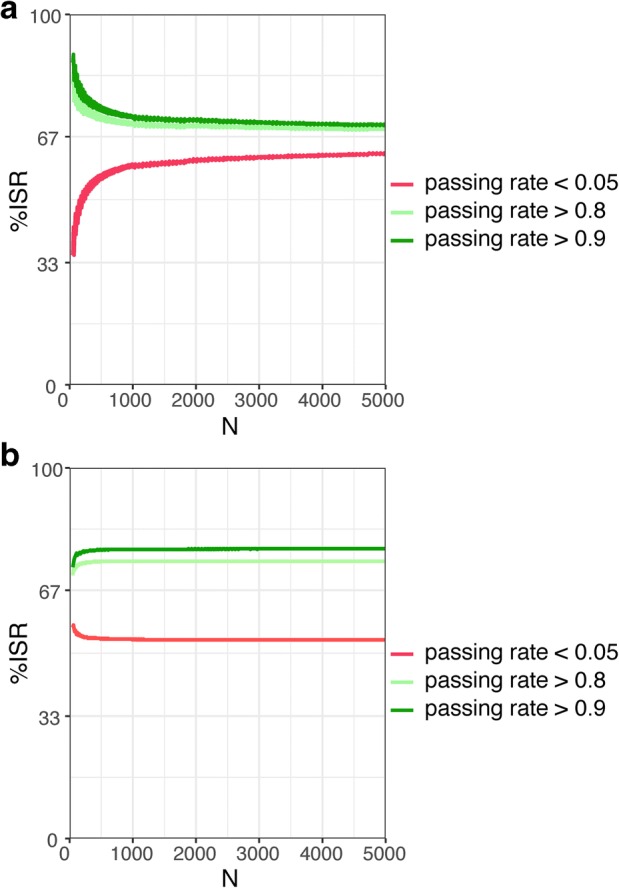
The %ISR needed to achieve a particular passing rate for different clinical study sizes using **a** fixed *n/N* ratio according to the current regulatory recommendations (5,7) and **b** a fixed *n* = 30. This figure confirms that the %ISR depends on the sample size (*N*) for the fixed *n/N*, but is nearly independent on the sample size (*N*) for the fixed *n*

The symbols and terms used in this manuscript are defined in Table I. Calculations and Figs. 1, 2, 5, and 6 have been generated in the R version 3.5.0 (25). For Figs. 1 and 2, we have selected a red-yellow-blue color scheme for seven diverging percentage difference classes in a colorblind-safe mode (26). Figures 3 and 4 have been generated in Microsoft Office Excel 2007.

**Table I:** Symbols and Terms Used

Symbol or term	Hypergeometric distribution	ISR test	Values tested and/or calculation method^a^
*N*	Size of the population	Study sample size—number of unique biological samples in a clinical study	(20), (50), 100, (200), (250), 500, 1000, 1500, 2500, and 5000
*n*	Number of the experiments	Number of ISRs	Fixed number: 10, 20, (30), 50, and 100 or fixed ratio: 5% · *N* (9), 7% · *N* (27), 10% · *N*, 10% · *N* for the studies with *N* ≤ 1000 and 100 + 5% · (*N*-1000) for the studies with *N* > 1000 (5, 7)
*K*	Number of successes in the population	Number of ISR pairs meeting %difference criteria if all samples from the clinical study have been analyzed	*K = p · N* *K* ∈ [0,1, …, *N*]
*k*	Number of successes in *n* experiments	Number of ISR pairs meeting %difference criteria observed in the reanalyzed samples	*k* ∈ [0,1, …, *n*]
*p =* %ISR	Success rate	true percentage of ISR pairs meeting %difference criteria (when all samples have been reanalyzed)	*p* ∈ [0; 100%] $p=\frac{\mathrm{K}}{N}\cdotp 100\%$
$\widehat{p}$ *=* %isr	Estimated success rate	The estimated percentage of ISR pairs meeting %difference criteria (when a portion of the samples has been reanalyzed)	$\widehat{p}$∈ [0; 100%] $\widehat{p}=\frac{\mathrm{k}}{n}\cdotp 100\%$
Passing rate	–	Probability of passing the ISR test	Calculated using the hypergeometric distribution passing rate ∈ [0; 1]
%difference	–	Percentage difference between the original concentration and the concentration measured during the repeat analysis	$\%\mathrm{difference}=\frac{\mathrm{repeat}-\mathrm{original}}{\mathrm{mean}}\cdotp 100\%\kern0.5em$(7)

^a^ *Values in brackets were used in selected tests only*

We have assumed that the population size (*N*) is typically between 100 and 5000 clinical samples, but in some calculations we have also used smaller sample sizes of 20 and 50. We have tested fixed *n/N* ratios as recommended at 5% (9), at 7% (27), and at 10% for comparison. We have also tested the reference two-step fixed *n/N* ratio recommended by the regulatory authorities: 10% up to 1000 samples and then 5% (5,7). A fixed number of 20 and 50 ISRs (*n*) is based on the previously reported assumptions (4,17). We have used the term “ISR pair” with reference to the related original and repeat values.

We have classified the bioanalytical methods as:

- non-reproducible (when the passing rate is not higher than 0.05),
- reproducible (when the passing rate is not less than 0.80),
- and quasi-reproducible (when the method does not meet non-reproducible nor reproducible criteria).

Note that the classification of the methods with the same *%ISR*—i.e., true percentage of the ISR pairs meeting %difference criteria when all samples are reanalyzed—may vary depending on *N* and *n.*

We have assumed that the test outcome for each ISR pair is dichotomous, i.e., belonging to one of two mutually exclusive categories: a success (if an ISR pair meets the %difference criteria) or a failure (if an ISR pair does not meet the %difference criteria). The sampling from a finite population has been carried out without replacement, that is why the hypergeometric distribution is more appropriate than the binomial distribution. The type I error (*α*) of 0.05 and power of 80% have been used as suggested by Rocci at al. (17). Additionally, we have used the power of 90%.

The acceptance criteria were in line with the FDA (7) and the EMA (5) recommendations on the bioanalytical method validation. The %difference (Table I) should be within ± 20% of the mean concentration for small molecules and within ± 30% for large molecules. The percentage of the ISR pairs meeting the %difference criteria (*%isr*) should be at least 67%.

## RESULTS

### Fixed Ratio of the Number of ISRs to Study Sample Size (*n*/*N*)

We have started from the scenario when *n/N* is fixed. This is in line with the current regulatory practice which uses a two-step fixed ratio depending on the clinical study size: *n/N* = 10% up to 1000 clinical samples and then *n/N* = 5% (5,7). We have also evaluated one-step fixed *n/N* at 5% (9), 7% (27), and 10%. For each *n/N*, we have plotted the passing rate vs. *%ISR* (Fig. 1) for selected *N* values.

Generally, all plots look similar regardless of *n/N* used (Fig. 1). Except for *N* of 100 and 200, the passing rates are comparable for all *n/N* ratios, including the passing rate (I) below 0.05 when *%ISR* ≤ 50, (II) over 0.80 when *%ISR* ≥ 75, and (III) over 0.90 when *%ISR* ≥ 80. For *N* ≤ 200—or for *n* ranging from 5 to 20—cumulative distribution functions look flatter. The results suggest that in this scenario non-reproducible and reproducible methods are better discriminated as the sample size increases.

### Fixed Number of ISRs (*n*)

Another approach to the ISR sample size is a fixed number of ISR pairs apart from the study size (4,17). So, we have fixed *n* at the following values: 10, 20, 50, and 100. We have combined each *n* with the selected *N* values and then we have plotted the passing rate vs. *%ISR* (Fig. 2).

Surprisingly and contrary to the fixed *n/N* (Fig. 1), nearly all curves for particular *n* are overlapping (Fig. 2). Only when both *n* > 20 and *N* = 100 the cumulative distribution functions look steeper (Fig. 2c, d, *N* = 100). Apart from these two exceptions—both far exceeding the currently recommended *n/N* of 10% (5,7)—the passing rate seems independent of the study sample size.

A more detailed look at the plots shows that when *n* increases, the cumulative distribution functions are steeper in the *%ISR* range of 50–70%. Thus, as could be expected, the reproducible methods are better distinguished from the non-reproducible ones when *n* increases. But to what extent should they be distinguished?

### To What Extent Should the Reproducible Methods Be Distinguished from the Non-reproducible Ones?

We needed two steps to answer this question. Firstly, we selected critical passing rates of ≤ 0.05 for non-reproducible methods (equal to the assumed acceptable type I error) as well as the passing rates of ≥ 0.80 and ≥ 0.90 for reproducible methods (equal to the typical values of power in bioequivalence studies (28)). As a reference, we calculated *%ISR* for each of the above passing rates (Table II) using the current regulatory ISR sample size (*n*) (5,7). Data presented in Table II and in Fig. 3 both confirm that using a two-step fixed ratio leads to different *%ISR* needed to get the same passing rate for different clinical study sizes (*N*). For example, in order to achieve the passing rate not exceeding 0.05 when *N* = 20, the method may have very low reproducibility (*%ISR* = 22.5%). But when *N* = 5000, then reproducibility may be quite similar to the acceptance criteria (*%ISR* = 62.3%). Thus, the current ISR sample size (*n*) leads to the acceptance criteria dependent on the clinical study size (*N*).

**Table II:** Theoretical %ISR Calculated for Different Passing Rates Using the Current Regulatory ISR Sample Size (5,7)

*N*	*n*	Passing rate ≤ 0.05	Passing rate ≥ 0.80	Passing rate ≥ 0.90
20	2	22.5%	87.5%	92.5%
50	5	35.0%	83.0%	89.0%
100	10	40.5%	75.5%	80.5%
200	20	50.2%	75.3%	78.8%
*300*	*30* ^*a*^	*54.1%*	*74.5%*	*77.9%*
500	50	56.0%	72.1%	74.5%
1000	100	58.8%	70.2%	72.0%
1500	125	59.9%	70.1%	71.8%
2500	175	61.3%	70.0%	71.4%
5000	300	62.3%	69.1%	70.2%

^a^ *Values in italics: the lowest n among the selected values to meet the following criteria: %ISR > 50% needed for the passing rate of 0.05 and %ISR < 75% enough for the passing rate of 0.80*

In the second step, we calculated %ISR for each of the critical passing rates using fixed *n* (Fig. 4, Table III). Contrary to the fixed *n/N* ratio, we observed a similar %ISR needed to get the same passing rate for different clinical study sizes (*N*). For example, when *n* = 30 is used to achieve the passing rate not lower than 0.80, for *N* = 100 the method should have the reproducibility of %ISR = 73.5%, while for *N* = 5000 the reproducibility of 1.4% higher (%ISR = 74.9%) is necessary. Thus, the fixed *n* leads to the acceptance criteria much less dependent on the clinical study size (*N*).

**Table III:** Theoretical %ISR Calculated for Different Passing Rates Using a Fixed Number of ISRs (*n*)

		%ISR (%)		
*N*	*n*	Passing rate ≤ 0.05	Passing rate ≥ 0.80	Passing rate ≥ 0.90
100	10	40.4	75.5	80.5
500		39.4	75.9	81.1
1000		39.4	76.1	81.2
1500		39.4	76.1	81.2
2500		39.3	76.1	81.3
5000		39.3	76.1	81.3
100	20	51.4	74.5	78.5
500		49.4	75.3	79.1
1000		49.4	75.4	79.3
1500		49.3	75.5	79.3
2500		49.2	75.5	79.3
5000		49.2	75.5	79.4
100	30^a^	55.4	73.5	76.5
500		53.8	74.7	77.9
1000		53.7	74.9	78.1
1500		53.6	74.9	78.1
2500		53.5	74.9	78.1
5000		53.5	74.9	78.2
100	50	58.4	70.5	72.5
500		56.0	72.1	74.5
1000		55.8	72.2	74.8
1500		55.6	72.3	74.9
2500		55.6	72.3	74.9
5000		55.5	72.3	75.0
100	100	66.4	66.5	66.5
500		59.2	69.9	71.7
1000		58.8	70.2	72.0
1500		58.6	70.3	72.1
2500		58.6	70.3	72.2
5000		58.5	70.3	72.3

^a^ *The lowest n among the selected values to meet the following criteria: %ISR > 50% needed for the passing rate of 0.05 and %ISR < 75% enough for the passing rate of 0.80*

**Fig. 7:**
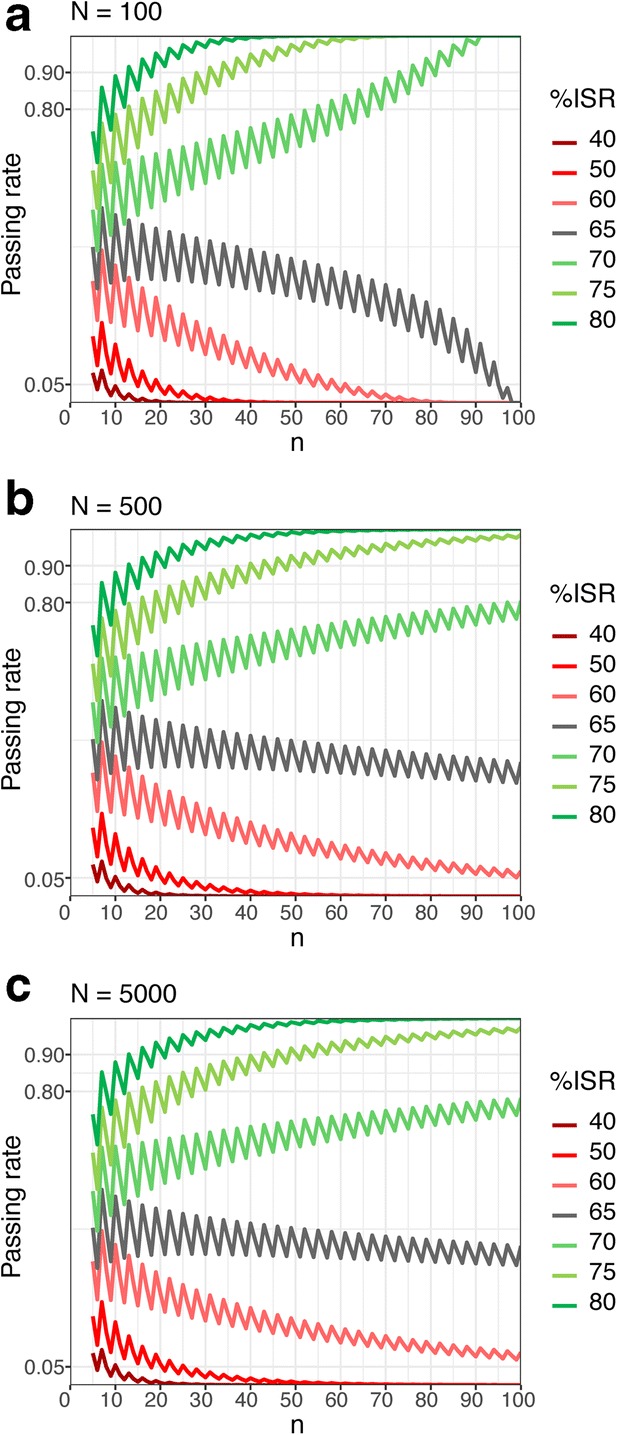
Passing rates for different fixed *%ISRs* as a function of the number of ISRs (*n*) for study sizes *N* of 100 (**a**), 500 (**b**), and 5000 (**c**)

As expected, the higher the *n*, the better reproducible methods are distinguished from the non-reproducible ones. But to what extent should they be distinguished? To figure out the answer, we added a pair of assumptions. Firstly, when 1 of every 2 samples meets the ISR acceptance criteria (%ISR = 50%), then the method is non-reproducible. Secondly, when 3 of every 4 samples meet the ISR acceptance criteria (%ISR = 75%), then the method is reproducible. For these assumptions, the hypergeometric distribution suggests that *n* = 30 is the right selection. For such *n*, the %ISR over 50% is needed to achieve the passing rate of 0.05. For the sample sizes of up to 5000, the %ISR below 75% is enough to get the passing rate of 0.80 (Fig. 5, Fig. 7 in Appendix I).

### How Does the Passing Rate Depend on %ISR?

To answer this question, we compared the fixed *n/N* ratio and fixed *n* concepts using %ISR needed to achieve particular passing rates. Figure 6 confirmed previous observations for different clinical study sizes. The %ISR depends on the sample size (*N*) for the fixed *n/N*, but is much less dependent on the sample size (*N*) for the fixed *n*.

## DISCUSSION

The goal of the ISR test is to confirm that a bioanalytical method is reliable (5,7). Thus, the probability of meeting the acceptance criteria should depend mainly—or even solely—on the bioanalytical method performance. Our new approach shows that this is not the case when the sample size is based on a fixed *n/N* ratio. The hypergeometric distribution revealed that the passing rate for a particular method performance (%ISR) is related to *N.* Surprisingly, this dependence is hard to observe for a fixed *n*.

Following the assumptions presented above, a fixed *n* = 30 seems to be the statistically right solution. The advantages of this approach over the current practice (5,7) are as follows:

(I). passing rates are much less dependent on *N* (Fig. 6); thus, the same performance means the same probability of passing the ISR test regardless of the clinical study size,
(II). it is simple and does not need any calculations in order to assess the ISR sample size,
(III). non-reproducible methods are better distinguished from the reproducible ones for *N* < 300,
(IV). fewer samples are analyzed for *N* > 300, which allows cost-effective and environmentally friendly bioanalysis in medium and large studies.

But, is the ISR sample size limited to 30 samples enough to detect problems with the method? A solely statistical approach may not be adequate to answer this question. An ISR test requires an appropriate experimental design (17,18). Samples for the test should include all phases of the study, different analytical batches, different subjects and samples stored for longer periods of time, and high and low analyte concentrations. An adequate representation of all variability factors is needed to detect problems with inhomogeneity of the sample as well as metabolism and stability issues. Thus, the proposed 30 samples is just an example, not a final solution. One should also note that the ISR test is not performed in vacuum. It is complemented by a system suitability test (SST) which confirms instrumental performance. Then, for each bioanalytical batch, the actual method performance is monitored by the calibration curve and QC samples. The suggested *n* = 30 is larger than (I) 20 ISRs initially proposed by Rocci *et al.* (17) and (II) a minimum of 20 samples proposed recently by FDA for bridging the data from multiple bioanalytical technologies (7). It is large enough to expect valid results of statistical analysis based on the normal distribution. It is also more comparable to the sample sizes of other validation tests. One may draw a comparison to the accuracy and precision evaluation: 5 samples of high (near maximum concentration) and low (in the elimination phase) concentration, each studied in 3 separate analytical runs, gives exactly 30 samples. Should the regulatory authorities find the simple approach of a fixed *n* = 30 unsuitable, it may be somewhat extended. One possibility is an adaptive method similar to our previous concept (12).

The calculations using the hypergeometric distribution are complementary to the published simulations (10,18,19), but they have significant advantages. The novel model uses %ISR to include both random (imprecision) and systematic (bias, metabolite conversion) errors. So, the inference for many combinations of precision and bias values is avoided, which greatly simplifies the problem. We have also managed to avoid making unnecessary assumptions—like the concentration range or the distribution of the concentration for individual results (10,18,19). Thus, our calculations presented here are better suited to the real datasets than the simulations. Another novelty is defining the results of each ISR pair as dichotomous (success or failure), what makes the calculations independent of the %difference acceptance criteria. Therefore, they are valid for both small and large molecules. The hypergeometric distribution is even more universal model as it may be also used to bridge data from multiple bioanalytical technologies.

One of the limitations of this paper may lie in the assumption that %ISR is a constant. One may argue that the true value of %ISR is unknown and a particular method may have somewhat different %isr in different studies. Due to many factors contributing to the variability, the %isr for a particular method in a single study may vary in time. Yet, by definition, %isr is an estimation of %ISR and we have limited the constant %ISR evaluation to a particular study. One may also suggest that a smaller number of ISRs will decrease a chance of locating problems, especially in larger studies. Each reanalysis increases the chance of unmatched results and figuring out their cause, but these opportunities are not always necessary to validate bioanalytical data (12). The hypergeometric distribution shows that an increasing number of samples over certain limit does not lead to better distinguishing of reproducible and non-reproducible methods (Fig. 6).

Bioanalysis is an important part of the drug development process. Finding an optimal ISR sample size needs appropriate balance between test ability to identify method-related problems and avoiding unnecessary analyses. The latter generate extra costs and delay research. So, creating a new performance standard for ISR may be one of the steps helping to provide patients with more timely and affordable access to new therapies, as suggested by the FDA (29). The reduction of the regulatory burden is especially anticipated for large studies, where hundreds of ISRs are being analyzed now, but even for standard-sized studies, savings may be considerable. This paper provides a basis to re-consider the current ISR sample size calculation based on the clinical study size (*N*). The proposed fixed *n* concept is not intended for instant practical application in the regulated bioanalysis, because it is challenging the current regulatory recommendations (5–7). However, the hypergeometric distribution has proved to be an appropriate model to help understand statistical relations between the accuracy and precision of a bioanalytical method vs. ISR test passing rates. So, the investigators interested in efficient way of validating repeatability of their non-regulated bioanalytical methods may use our approach instantly. We hope that this model may help regulatory bodies to implement more statistically rationalized and risk-controlled ISR methodology. It may be the right time for change, as the ICH is currently developing its global bioanalytical method validation guideline (30). The acceptance of the idea might need more detailed comparison of all the models used for ISR evaluation, thus future studies on this topic are invited.

## CONCLUSION

The hypergeometric distribution is an appropriate model to understand and optimize ISR sample size better. Our study revealed that the passing rates are currently related to clinical study size*.* Interestingly, the passing rates are much less dependent on clinical study size when a fixed number of ISR samples are used; therefore, we propose to use a constant number of samples, e.g., 30, for ISR for all studies. This paper provides a basis to re-consider ISR methodology and implement a more statistically rationalized and risk-controlled approach.
